# The social environment and cognitive aging over the life course: laying out critical concepts and research gaps

**DOI:** 10.1002/alz.71648

**Published:** 2026-07-13

**Authors:** Margaret T. Hicken, Reed DeAngelis, David Rigby, Lindsey Burnside, Sara Adar, Sarah Burgard, Brigette A. Davis, Rachel Donnelly, Jessica M. Finlay, Anjum Hajat, Jinkook Lee, Brea L. Perry, Lorna E. Thorpe, Debra Umberson

**Affiliations:** ^1^ Institute for Social Research University of Michigan Ann Arbor Michigan USA; ^2^ Department of Epidemiology University of Michigan Ann Arbor Michigan USA; ^3^ School of Medicine Washington University in St. Louis St. Louis Missouri USA; ^4^ Department of Sociology Vanderbilt University Nashville Tennessee USA; ^5^ Department of Geography University of Colorado Boulder Boulder Colorado USA; ^6^ Department of Epidemiology University of Washington Seattle Washington USA; ^7^ Department of Economics University of Southern California Los Angeles California USA; ^8^ Department of Sociology Indiana University Bloomington Bloomington Indiana USA; ^9^ Department of Population Health NYU Grossman School of Medicine New York New York USA; ^10^ Department of Sociology University of Texas at Austin Austin Texas USA

**Keywords:** neighborhood, residential context, social connections, social environment, work context

## Abstract

The *social environment* refers to our interpersonal relations, workplaces, and neighborhoods, towns, or cities in which we live. A growing literature indicates that social environments are related to cognitive aging and risk of Alzheimer's disease and related dementias (AD/ADRD). Still, relatively little is known about *how* social‐environmental exposures affect cognitive function over the life course and into older ages. Addressing this limitation, our paper outlines key features of the social environment and recommends priority areas of research on the social environment and cognitive aging. We divide our discussion into three subdomains: social connections, residential context, and work context. We then identify important gaps in the conceptual and empirical literature before outlining avenues for future research to strengthen our understanding of how social‐environmental exposures over the life course link with cognitive function and AD/ADRD risk in late life.

## Highlights

- The social environment is linked to AD/ADRD risk, but key concepts and measures require clarity.
- Key features of the social environment linked to life course cognitive aging are discussed.
- Focal gaps in the conceptual and measurement literature are discussed.

## 1 INTRODUCTION

In broad terms, the *social environment* “includes the groups to which we belong, the neighborhoods in which we live, the organization of our workplaces, and the policies we create to order our lives”^1^ and further the “cultural milieus within which defined groups of people function and interact.”^2^ In other words, the social environment can be considered as the interrelated composite of three domains: (1) *social connectedness*, including our ties to other specific people where we live, work, and play and to broader communities or societies; (2) *residential context*, including features of our neighborhoods, towns, and cities that facilitate or limit, for example, social interaction (i.e., “social infrastructure”) and work opportunities; and (3) *work context*, including workplace conditions, occupations, and even the broader labor market dynamics that facilitate and limit not only economic and material resources but also our social connections.

**FIGURE 1:**
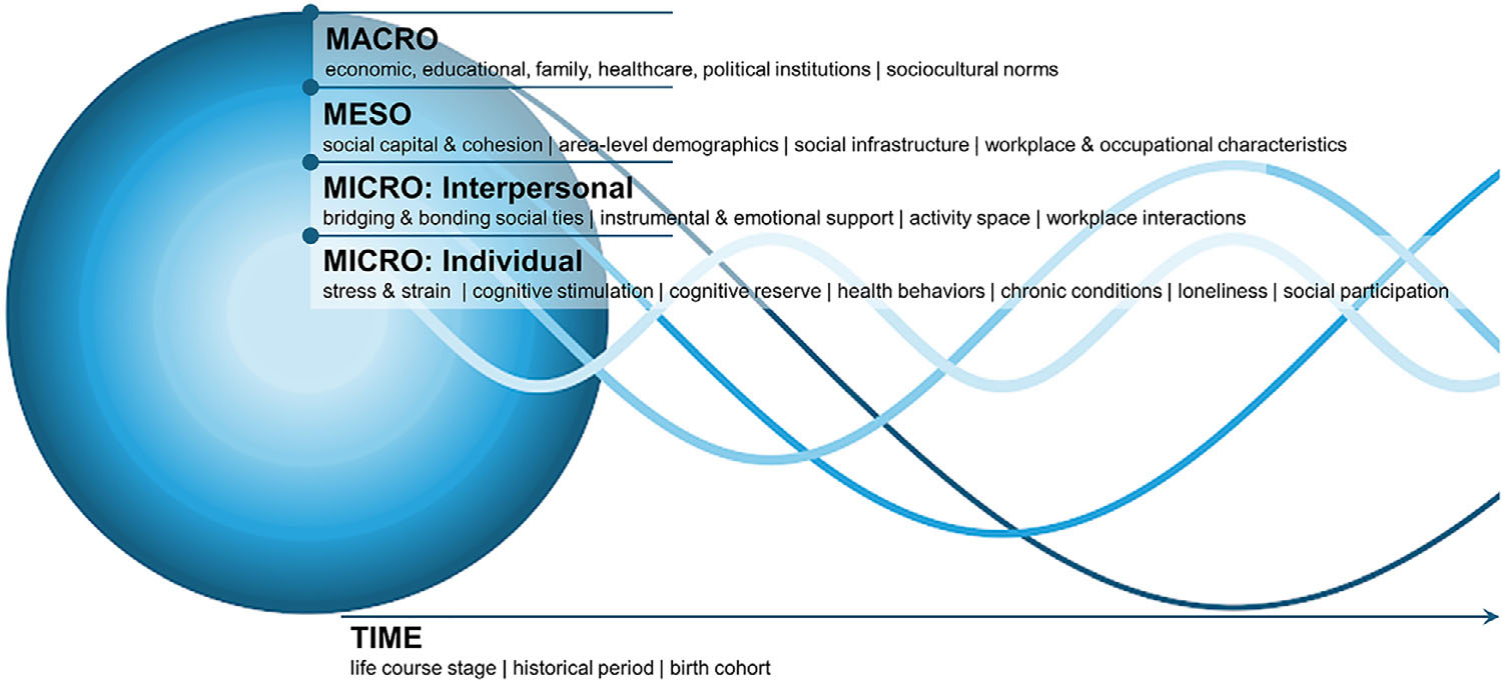
Conceptual diagram depicting dynamic features of social environment in relation to macro‐level forces and individual‐level risk of AD/ADRD over the life course. Note that this figure represents features of the three primary domains of the social environment (i.e., social connections, residential context, and work context) at different ecological levels. It is posited that features of the inner levels are shaped by the outer levels. For example, the individual‐level health behavior of physical activity is thought to be shaped by the norms of one's social network, the availability of social infrastructure such as walking pathways, and community physical activity organizational programs. The color gradients of the ecological levels represent the reality that not every feature of the social environment fits neatly into a single layer but rather accretes in dynamic and complex ways around the micro level (e.g., social cohesion may be perceived by the individual and depend on mutual interpersonal relationships, but it is an emergent property of a community). The sine waves that flow from each ecological layer tangent represent the dynamic and interactive flows across individual life course stages (e.g., quality of childhood education, adulthood workplace characteristics, health behaviors) within social connections, broader residential and work contexts, and, finally, society as a whole. The compilation of articles outlined in Table 1 provides information to accompany this figure.

Our approach is grounded in frameworks that conceptualize social‐environmental exposures as “meso‐level” community and “micro‐level” interpersonal forces that may link “macro‐level” institutional laws, policies, regulations, and sociocultural norms (e.g., importance of paid work, views on family structure, individualistic vs communalistic orientation) to “micro‐level” individual and proximate health and behavioral risks that might be linked to cognitive health over the life course (Figure 1). While most conceptual and empirical research on cognitive health and AD/ADRD specifically focuses on proximate, individual‐level risk factors,^3^ a growing literature also suggests a critical role for many aspects of the social environment in shaping these risk factors over the life course. For example, contextual aspects of neighborhoods (which are clusters of people living in a place with shared physical or symbolic boundaries) have been linked to dementia risk factors like cardiovascular disease,^4^, ^5^ hypertension,^6^, ^7^ diabetes,^8^, ^9^ and numerous health behaviors.^10^, ^11^ Likewise, various features of paid work have been linked with heart disease and stroke,^12^ as well as anxiety and depression.^3^, ^13^ Reports suggest that social isolation (the paucity or lack of social relationships) is related to the risk of morbidity and mortality at similar or greater magnitudes than other well‐known risk factors like diabetes, alcohol consumption, physical inactivity, and exposure to air pollution.^14^, ^15^, ^16^ The meso‐level and micro‐interpersonal‐level domains of the social environment, because they may shape the more proximate AD/ADRD risk factors, may serve as efficient and effective primary and secondary prevention intervention points (preventing dementia or cognitive impairment development and minimizing the impact of these conditions, respectively).^17^ In other words, they may point to the factors that shape population risk over the life course beyond the individual‐level risk.

However, before we can point to these potential intervention points, we need to clarify the conceptual features of the social environment (generally drawn from multiple social sciences) that are plausibly related to the risk of AD/ADRD. With conceptual clarity particularly around their defining characteristics, we can better understand the strengths and limitations of our data and measures so that the field can continue to develop the empirical evidence needed to address both overall risk and social inequalities in risk. The purpose of this introductory overview is to (a) bring together the primary domains of the social environment into a single discussion to guide new research that might better reflect the complicated and complex nature of AD/ADRD risk over the life course; (b) facilitate the navigation of a conceptual and empirical literature that is sparse in some areas (e.g., third places or those places that support social connections that are not home or work) and dense in others (e.g., social isolation) by providing key recommended reading for those who wish to embark in a certain area of the social environment; and (c) identify persistent gaps in the literature and recommendations for the next empirical steps. Because the literature generally focuses on one domain (e.g., residential context) or one feature (e.g., neighborhood poverty, social infrastructure – the infrastructure that supports social connections), we describe each of the domains in turn but bring all of the features together in Figure 1, Table 1, and our discussion of future directions for empirical research. Within each domain, we outline the concepts and potential pathways to life course AD/ADRD risk; in Table 1, we provide article compilations to provide multidisciplinary overviews of the concept and measures; then, in Figure 1, we highlight how the concepts likely fit together in a life‐course ecological framework. We start with a discussion on social connectedness, as it is thought to serve as an important mechanism linking both residential and work contexts to cognitive aging over the life course.

**TABLE 1:** Recommended reading on key concepts and measures in social environment.

**Life course** The life course framework^18^, ^19^ has been conceptualized in multiple disciplines and integrates not only the lifespan but micro‐individual‐level periods in the lifespan that may be more strongly related to macro‐ and meso‐level forces. Age‐period‐cohort^20^, ^21^, ^22^ is a concept drawn from demography that outlines the interrelated nature of the micro‐individual‐level life course (i.e., age), the period in history in which one lives at different points in the life course, and the birth cohort from which people set out the trajectory of their life course. These three factors are intertwined in a dynamic way over time.
**Social connectedness** Social capital^23^, ^24^, ^25^, ^26^, ^27^, ^28^ has been conceptualized through the lens of direct interpersonal exchanges within a social network and through the lens of a meso‐level community property that emerges from a collection of individuals. These two lenses have some, but not complete, conceptual overlap, which has implications for measurement either through area‐level data or individual‐level surveys. Social cohesion^28^, ^29^, ^30^ is related to social capital, and, though it is considered a meso‐level property of communities, challenges arise when it comes to connecting this concept with the data, which are often captured through individual‐level surveys. Social isolation,^31^, ^32^, ^33^, ^34^ as well as loneliness,^15^, ^31^, ^34^, ^35^ have been long‐studied with numerous measures, each with utility and limitations, depending on the empirical need. This compilation includes a scoping review of measures with recommended practices.
**Residential context** Activity space,^36^, ^37^, ^38^ drawn from geography and sociology, may better reflect the reality of the ways in which residents navigate everyday life, but it has not been empirically explored widely in relation to life course cognitive aging. The two primary conceptualizations use GPS data but build on different measures. Place‐based social organizations,^39^, ^40^, ^41^, ^42^ like other parts of our social infrastructure, are designed to facilitate social connections, to specific others or to a larger whole. “However, the regular programs and activities at the core of place‐based community organizations make them distinct from other forms of social infrastructure.”^39^ We include an overview of micro‐individual‐level participation in relation to cognitive aging as a related concept. Residential demographic characteristics^40^, ^43^, ^44^, ^45^, ^46^, ^47^, ^48^ have been long studied, but they face both conceptual and measurement challenges. This compilation provides an overview of these issues and potential remedies, including approaches for categorical and ordinal indicators. Rural social infrastructure^49^, ^50^, ^51^, ^52^, ^53^ has been more richly conceptualized, beyond the rural–urban dichotomy, in regional planning and geography literature; this richness is now gradually being integrated into health studies. The gradient of rural and small‐town context presents unique challenges to infrastructure measurement as communities adapt to investments creating social places from spaces that would not be used in similar ways in urban settings. Urban social infrastructure^40^, ^43^, ^54^, ^55^, ^56^, ^57^ conceptualizations have changed over time; the reader must take care to note the publication date. This compilation includes reviews that can orient the new reader to these changes and scoping reviews that discuss current measurement approaches.
**Work context** Occupational complexity^58^, ^59^, ^60^, ^61^ has been conceptualized in different social sciences as work that requires cognitive engagement with people, data, or things. This categorization was developed as a typology before substantial changes in computer technology and AI, however. Precarious employment^62^, ^63^, ^64^ has been conceptualized in sociology but more recently adapted to population health and aging. There are ongoing discussions around the defining characteristics of precarious employment, with implications for measurement, as discussed in this compilation. Workplace, occupational, and job characteristics^61^, ^65^, ^66^ have been discussed across the social sciences. Occupation and job are sometimes used interchangeably; however, the former is a broader categorization of jobs. Job characteristics are often measured using occupational class characteristics derived from the US Bureau of Labor Statistics, which is why these terms are often used together. Work‐related psychosocial stressors^17^, ^67^, ^68^ have also been discussed across the social sciences with numerous frameworks to explain the link between stressor exposures and the experience of strain.

*Note*: With each concept, the recommended reading is compiled in sets to provide the reader with a thorough introduction to the concept, its relation to cognitive aging (if available), and current measures.

## 2 SOCIAL CONNECTEDNESS

Social connectedness is a broad, multifaceted concept that transcends residential and work contexts and includes the (a) quantity and structure of our micro‐interpersonal‐level social relations and interactions, (b) resources derived from those social relations, whether to specific others or to a larger community, the latter of which can be conceptualized as a meso‐level property of communities, and (c) balance of positive and negative emotions stemming from social relations.^69^, ^70^ Social connectedness thus includes both objective (e.g., number of ties, living arrangements) and subjective (e.g., loneliness) features. Social connectedness is thought to have critical, reciprocal links with health across the lifespan; our social relations can offer us emotional and instrumental support to cope with stressors, promote our sense of self and belonging, provide opportunities for cognitive engagement, and encourage healthy behaviors.^71^, ^72^, ^73^, ^74^, ^75^, ^76^, ^77^ Conversely, a robust literature suggests that social isolation and relationship strain or stress are major risk factors for morbidity and mortality.^14^, ^15^, ^35^, ^78^

Social connectedness is operationalized in numerous ways. Indeed, entire bodies of work have developed across multiple disciplines that analyze social connectedness at different levels, ranging from individuals to dyads to social networks to entire societies.^24^, ^69^, ^78^, ^79^ Here, we limit our focus to three aspects of social connectedness that have overlapped with research on health and cognition: social networks and ties, social isolation/loneliness, and social capital, cohesion, and its related concepts.^25^, ^69^, ^78^, ^80^

Social networks encompass our family, friends, coworkers, neighbors, and anyone we interact with regularly. Social networks also reflect the structure of our social ties, including whether and to what extent network members are connected to one another.^25^, ^81^ Scholars typically employ survey methods and egocentric network analysis to assess a respondent's number of social ties, the structure of relations among their ties, and the quality or functions of relationships, such as the extent to which their ties provide resources like information, financial assistance, or emotional support.^82^, ^83^ Emerging evidence suggests that people with expansive social networks composed of ties that cut across or link diverse social groups (i.e., “bridging ties”) tend to exhibit better cognitive function, relative to peers with networks consisting of fewer and more close‐knit (i.e., “bonding”) ties. The causal processes underlying these patterns are complex and likely bidirectional. On the one hand, some posit that people with diverse social networks tend to be exposed more frequently to novel social stimuli (e.g., diverse ideas, information, and speech patterns), which can enhance brain function through cognitive stimulation and complex processing.^25^, ^81^ On the other, people with better cognitive function may have greater capacities for maintaining large and diverse social networks in the first place.^84^, ^85^, ^86^ Our social networks also expose us to different health norms, behaviors, and information that could have both positive and negative effects on cognitive health. For instance, while social ties can reinforce healthy norms like regular doctor visits, dieting, and exercise, our networks can also promote risky behaviors like smoking, binge drinking, and sedentariness.^87^

*Social isolation* refers to the quantitative lack of social relationships or interactions.^78^ Common indicators of social isolation include living alone, minimal or no social ties, and infrequent contact with other people.^15^, ^78^ Thus, social isolation is typically measured by asking people to report counts of relationships, frequencies of interaction, whether they are married or partnered, and/or whether they live alone.^78^, ^88^, ^89^ Studies suggest, for example, that living with a romantic partner may mitigate negative effects of stress on cognitive function.^25^, ^81^ Indeed, recent evidence from the Health and Retirement Study (HRS) indicates that participants who score higher on scales of social isolation exhibit signs of accelerated cognitive decline^90^ and aging‐related biomarkers.^91^ Some of these patterns appear to be related to proximate health risk factors reported more frequently among socially isolated persons, including smoking, drinking, sedentariness, sleep disturbances, and depressive symptoms.^91^

Still, people perceive social relationships differently,^89^ and these perceptions also may matter for health.^15^ *Loneliness* describes the subjective experience of insufficient social connectedness, reflected by dissatisfaction with the quantity and/or quality of social connections and interactions.^15^ Although social isolation and loneliness are both linked to poor health, mortality, and cognition specifically, they do not correlate strongly with one another.^15^, ^92^, ^93^ For example, some report that social isolation is more strongly linked to mortality, while loneliness is more strongly linked to dementia onset.^94^, ^95^, ^96^, ^97^ This suggests both dimensions capture unique facets of social disconnectedness and are not interchangeable.

*Social capital* broadly refers to the material, informational, and psychosocial resources people can access through social relations.^83^ Because social capital operates via interpersonal connections, the resources can be thought of as assets of individuals, groups, or even entire communities and societies. Resource exchanges are facilitated by trust, reciprocity, streamlined information channels, and social norms and sanctions that promote solidarity.^98^, ^99^ The presence of such factors (i.e., social norms, trust) within a group or society may be described as *social cohesion*.^29^, ^98^ Social cohesion is an emergent property of groups, though it is usually measured via individual attitudes and beliefs, which are then aggregated to some predefined group level (e.g., neighborhoods, schools, or entire nations).^80^, ^100^, ^101^, ^102^ Conceptually related to social capital, group social cohesion can also confer health benefits for individual members by regulating healthy behaviors (e.g., smoking abstinence) and facilitating social exchange, but it can also operate through feelings of collective efficacy and belonging.^98^, ^103^, ^104^, ^105^ For example, studies have found that people tend to perform better on certain cognitive tests if they also live in areas where they perceive a stronger sense of trust, available support, and belongingness among residents.^106^, ^107^, ^108^ Diminished psychological distress appears to account for some of these patterns.^108^ However, studies in this area often measure cognitive function and community cohesion/capital using individual‐level survey instruments, which raises concerns over confounding by other unobserved person‐level characteristics (also known as “common method bias”).^98^

## 3 RESIDENTIAL CONTEXT

Evidence suggests where we live can affect our cognitive health over the life course. For instance, dementia incidence and prevalence are higher in the southern United States (US) relative to other US regions.^109^, ^110^ Adults living in rural areas also show greater risks of cognitive impairment than their urban counterparts.^111^, ^112^ To be sure, selection effects can partially account for these patterns, meaning people who already exhibit similar cognitive functioning may also tend to migrate to similar areas.^113^ Still, features of the residential context may independently compromise or support healthy cognitive aging.^114^ Shaped by macro‐level forces such as housing markets and regional and local economic conditions, meso‐level public and private investments in local social infrastructure may then shape the nature of our micro‐interpersonal‐level social connections both stressful and supportive^115^; our micro‐individual‐level opportunities for and barriers to both salubrious (e.g., physical activity) and harmful (e.g., smoking, alcohol use) behaviors as well as other forms of cognitive stimulation (e.g., libraries, museums)^114^, ^116^; and the nature of macro‐level employment opportunities, meso‐level workplace characteristics, and micro‐individual‐level job stress and strain (Figure 1). In what follows, we focus on two broad and interrelated components of residential contexts: (1) area‐level sociodemographic characteristics and (2) social infrastructure.

Area‐level sociodemographic characteristics refer to population compositions within spatial boundaries or administrative units, such as neighborhoods and counties, and are among the most general descriptors of residential contexts. People with similar sociodemographic characteristics tend to cluster within similar neighborhoods, cities, and towns across the United States. The reasons for these patterns are deeply historical and complex and, thus, beyond the scope of this introductory overview (for more information, see Rothstein,^117^ for example). Nevertheless, evidence suggests geographical sociodemographic characteristics are robustly related to health generally and cognitive aging more specifically. For example, older adults who live in zip codes with higher levels of socioeconomic adversity exhibit not only greater risks of dementia^118^ but also mortality following an AD/ADRD diagnosis.^119^ Further, residents living in areas of concentrated poverty report faster declines in episodic memory relative to their peers living in areas of more socioeconomic privilege.^120^

From the aforementioned studies, the reasons for these spatial patterns in measures of cognitive aging are not clear and may range from individual‐level resources (e.g., income, healthcare access) to community‐level resources (e.g., parks, sidewalks, grocery stores). Thus, relying on area‐level sociodemographic indicators like the percentage of residents living below the poverty line, age distributions, or racial‐ethnic compositions precludes recommendations for specific interventions. Still, one major advantage of these measures is their relative ease when creating and linking to studies of cognition, such as population surveys with cognitive assessments or healthcare records with dementia diagnoses. Additionally, such measures can proxy a wide range of residential exposures with complex consequences for cognitive aging, which may be otherwise difficult and time‐consuming to capture through more granular measures. For example, neighborhood‐level poverty rates or other indicators of socioeconomic adversity likely reflect public and private correlates like home values and household wealth, access to food outlets, and availability of spaces amenable to physical activity and cognitive stimulation. Thus, examining associations between individual cognition and area‐level indicators like these can provide general insights into the role of residential contexts for cognitive aging and AD/ADRD risk.

**FIGURE 2:**
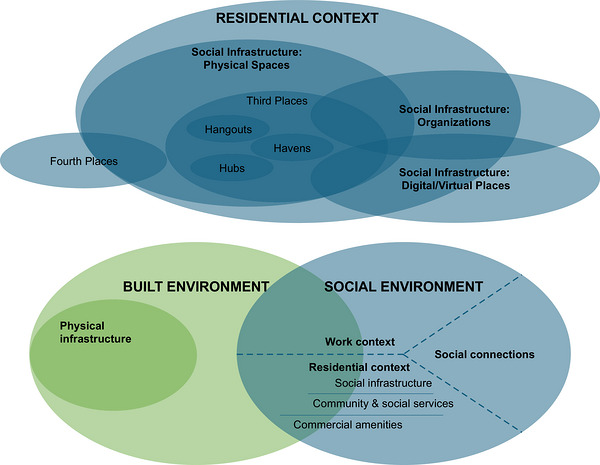
(A)Conceptual diagram of relations among dimensions of social infrastructure and local residential context. (B) Conceptual diagram of relations among built and social environments. Note that, informed by prior literature, this figure depicts the relations between dimensions of social infrastructure and local residential contexts (i.e., neighborhoods, towns, or cities). Three overlapping types of social infrastructure are physical spaces, organizations, and online spaces. Social infrastructure can also contain combinations of all three types (e.g., online neighborhood groups). “Third places” are specific types of social infrastructure outside of home (first place) and work (second place). Research on third places and cognitive aging typically focuses on physical locations (e.g., coffee shops), with some discussion of online spaces and organizations. Third places have been loosely categorized based on the types of social connections they foster. “Hubs” are third places that usually facilitate casual social interactions among diverse people. “Havens” tend to facilitate more intimate interactions and bonds among similar, like‐minded people. “Hangouts” typically support ambient social contact (e.g., “people watching”). “Fourth places” are transitional spaces that can also facilitate casual social interaction (e.g., vestibules, bus stops, commuter trains). Unlike third places, fourth places are typically not designed for the explicit purpose of fostering social interaction. Hubs, havens, and hangouts can also be part of the same organizations or digital/virtual places. Hangouts also have uses that overlap with some fourth places. Fourth places, social organizations, and social online spaces can move beyond the local residential context to a broader residential context (e.g., national or international region). In Figure 2A, residential context is depicted as the local environment with features of the social infrastructure and fourth places spanning beyond the local environment. In Figure 2B, residential context is depicted as “global,” as opposed to local, meaning that the context includes neighborhood, towns, and broader regions and country. All these components are depicted as distinct entities for simplicity.

Social infrastructure refers to the constellation of physical or digital spaces and organizations that facilitate social connectedness to specific others or to broader communities.^54^, ^55^, ^56^, ^57^ Figure 2 conceptualizes intersections among residential contexts and components of social infrastructure. We note that definitions and typologies of social infrastructure are still evolving.^56^, ^57^ For instance, one review reported that frameworks of social infrastructure have included features as diverse as housing, transportation, healthcare, and education. Recently, however, social infrastructure more recently has been conceptualized specifically as spaces that support social connectivity,^56^ with other types of the built environment, physical infrastructure, community and social services, and commercial amenities conceptualized as designed primarily for different purposes, although they may support social connection as well. The social connections that may be supported through social infrastructure range from interpersonal connections facilitated by coffee shops and recreational clubs, for example, to the broader connection that people may feel to their community as a whole that may be facilitated by, for example, libraries, museums, and faith‐based organizations. While most of the literature focuses on the social connections facilitated by social infrastructure, they may provide opportunities for direct cognitive stimulation and promote both salubrious and harmful health behaviors. Similarly, while most of this literature focuses on specific types of places (likely due to data availability), organizations, both placed‐based and online‐based, may facilitate social connections, cognitive stimulation, and health behaviors. Therefore, we focus our discussion on third places here and provide introductory reading in Table 1 for organizations.

*Third and fourth places* are considered types of physical social infrastructure designed to facilitate varying kinds of social interactions or connections or, as more recent discussions point out, to connect different types of physical spaces. Succinctly put, “third places are … physical locations outside of the home (first place) or workplace (second place) that facilitate social interaction, community building, and social support.”^121^ To be sure, many spaces that become third places were not designed only for social connections; for example, commercial spaces such as coffee shops or civic spaces such as walking paths were designed for other primary purposes but often consider social life in their design. Third places can be categorized by the types of social connections they foster. For instance, *hubs* are places where diverse groups of people can gather, visit, and interact in casual settings to form “weak,” or peripheral, bridging social ties. Other third places, known as *havens*, allow people with common interests to gather and interact in more intimate settings to form stronger bonding social ties. Finally, *hangouts* are spaces where diverse groups of people can visit without having to interact at all.^122^ Fourth places are transitional spaces (e.g., vestibules), edges that connect places (e.g., bus trips), and other “in‐between” spaces with varying degrees of social contact.^123^ Fourth places include spaces where people can be alone but not isolated, such as sites for “people watching” and other forms of ambient social contact.^124^ Though conceptually similar to hangouts, social interactions occurring within fourth places are usually unintentional. Unlike third places, which are typically conceptualized as components of local residential contexts, fourth places also connect people to different neighborhoods, towns, or even regions of the country or world. Though relatively new, the concept of fourth places entails similar notions of transportation and walkability originally included in rudimentary third place typologies.

## 4 WORK CONTEXT

Paid work is a major, multidimensional physical and psychosocial context in which most adults spend a major portion of their waking hours; it structures our daily lives, determines the cognitive and physical tasks in which we engage, allows for diverse social ties, and provides financial and other resources.^125^, ^126^ Research suggests important links between cognitive function and the quality, stability, and duration of employment,^127^, ^128^ cognitively stimulating work,^58^, ^129^ and work‐related stressors and physical hazards.^13^, ^130^, ^131^ In light of this research, we organize our discussion of work and cognitive aging around four overlapping themes: (1) occupational complexity, (2) occupational stressors, (3) material resources, and (4) non‐employment (i.e., job loss and retirement). In addition to considering each of these themes independently, one can conceptualize both occupational stressors and material resources through the lens of *precarious employment*, a construct characterized by job insecurity, low wages, few fringe benefits, and limited rights and protections.^62^

Occupational complexity (i.e., substantive complexity) refers to the degree of cognitively stimulating responsibilities at work, including levels of critical thinking and subtle reasoning required to make decisions and perform tasks.^132^, ^133^ Engaging in cognitively stimulating work has been shown to protect cognitive health across the life course.^58^, ^134^ Occupational complexity is often operationalized across three dimensions, by measuring the complexity of work with data, people, and things. Of these three dimensions, complex work with people is associated with decreased odds of cognitive decline and dementia, with weaker or mixed evidence for an association with complex work with data or things.^59^, ^135^

Occupational stressors (i.e., job stress, job strain) come in many forms. For instance, job strain occurs when employees receive excessive demands from their employers and are deprived of decision‐making power over how to meet these demands.^67^ Workers experience role strain when work responsibilities overwhelm and compromise performance in other social roles (e.g., family).^136^, ^137^ Workers can also experience job insecurity whenever they feel powerless to secure stable employment.^138^ Occupational stressors can also manifest as physical hazards like extreme temperatures and toxicants. All of these stressors have been linked with poor cognitive function and other AD/ADRD risk factors.^3^, ^12^, ^13^, ^130^, ^139^, ^140^

Paid work is the primary source of income, health insurance, and retirement benefits for most workers and their families.^125^, ^141^ People with more of these resources typically exhibit better cognitive function than their disadvantaged peers.^142^, ^143^ This is likely because people with more financial resources, for instance, are better situated to avoid chronic financial stress^144^ and to access neighborhoods with healthful amenities like greenspace and clean air.^145^

Unemployment is differentially related to dementia risk and depends on the life course timing, duration, and reason. Unemployment may limit the opportunities for cognitively challenging activities and social interaction, disrupting the cognitive stimulation that is critical to accumulating and maintaining cognitive reserve.^146^ Unplanned unemployment may also negatively affect access to health‐promoting resources and – especially early in one's work history – employment gaps may “scar” work trajectories and future earnings, with cumulative effects on health.^126^ Loss of the status and material resources associated with work may also lead to increased psychosocial stress, with consequences for cognitive health. Retirement may relate to declines in cognitive function because it removes the cognitively engaging aspects of work (e.g., cognitive stimulation, social interaction). However, there is evidence that retirement can have a positive effect on cognitive health, especially when work is physically demanding.^147^, ^148^ Therefore, the links between unemployment and cognitive health depend upon the reason for unemployment, the types of social and cognitive stimulation engaged during periods of unemployment, and other financial resources available to workers during these periods.

## 5 FUTURE RESEARCH PRIORITIES

In the preceding sections, we outlined the three primary domains of the social environment, describing their conceptualized pathways to cognitive aging and AD/ADRD risk over the life course. We now consider some empirical challenges to clarifying these mechanistic pathways. Our recommendations for addressing these challenges are threefold, to develop (1) more layered, multi‐dimensional, transdisciplinary life course theoretical frameworks that outline the potential pathways linking exposures to proximate risk factors and cognitive aging; (2) more granular conceptualizations of social‐environmental exposures; and (3) new measures that better reflect underlying concepts.

### 5.1 Expanded frameworks

Reflecting the extant literature, our discussion so far has treated social connectedness and residential and work contexts as somewhat distinct aspects of the social environment. Yet these components intersect in multiple ways, and we provide just a few examples. Research suggests the transition to retirement can precede declines in cognitive function, partially due to a loss of work‐related social ties. Indeed, studies have linked retirement with increased reports of social isolation and loneliness.^149^ These changes appear to relate mostly to a loss of bridging or peripheral social ties, rather than strong ties to close friends or family.^149^, ^150^ Moreover, engaging with social ties and groups appears to be more strongly related to cognitive function *before* retirement.^151^ Together, these results paint a complex picture of the linkages between work, social connections, and cognitive aging. Given that changes in social network compositions may predict cognitive aging outcomes,^24^ more research is needed to understand whether and how work affects cognitive function via social network ties.^59^

Research in the United States also tends to operationalize social connectedness as a property of individual social networks. But studies in other countries point to the importance of social infrastructure as a facilitator of social connectedness.^152^, ^153^ For instance, older residents of disadvantaged neighborhoods tend to report stronger feelings of isolation and faster cognitive decline, relative to their advantaged peers.^43^, ^154^ Parallel evidence suggests these patterns could be explained by inequalities in various public and private amenities, including different types of social infrastructure.^155^ Nevertheless, more research is needed to understand the complex linkages between residential sociodemographic characteristics, social infrastructure, and resident social networks and cognitive aging outcomes.

More work is also needed to understand how aspects of the social environment interact with other parts of the environment or context, such as physical environments and policy contexts, to affect cognitive aging. For example, opportunities to form social connections can be affected by elements of the natural and built environments, including extreme weather events, pollution, and access to public greenspaces, to name just a few.^156^, ^157^, ^158^ Further, policies related to social welfare, education, and labor markets can also impact local job opportunities and potentially moderate the links between work environments, social networks, and cognition.^159^, ^160^

Cognitive aging is also a lifelong process. Social‐environmental exposures early in the life course can ultimately influence processes of cognitive aging into adulthood and old age. For instance, residential contexts in childhood can limit opportunities for schooling and eventual access to higher education in young adulthood.^161^ Differences in educational attainment, in turn, have been linked to social inequalities in cognitive aging,^162^, ^163^ with some evidence suggesting a direct causal effect.^164^, ^165^ Educational attainment not only related to later stable employment and earnings, but research also suggests that it is related to employment quality, marked by meso‐level benefits such as healthcare, retirement plans, and paid leave and micro‐individual‐level job satisfaction.^166^ These and other features of occupational quality have been linked to morbidity risk factors for cognitive decline.^167^ Future research should test life course frameworks (see Table 1 for introductory reading) with longitudinal data to identify whether and how the social environment shapes risk factors for cognitive decline across the life course. For example, several high‐quality, national longitudinal studies have followed large cohorts of children, adolescents, and middle‐ and older‐age adults for several decades, documenting changes in their education, residential locations, occupations, social relationships, and health and cognitive function. It is important, however, to move beyond correlations to better understand potentially causal mechanisms, the directionality of associations, and the impact of spatial selection. Recent reviews outline different approaches to coordinated data analysis and methods to address time‐varying confounding and spatial selection (see Brenowitz et al.,^113^ Nichols and Hayes‐Larson,^168^ Nichols et al.,^169^ and Graham et al.^170^). Datasets like these, along with sophisticated modeling approaches, offer unique opportunities to study how different social‐environmental exposures shape cognitive aging trajectories across the entire human lifespan.^17^, ^171^, ^172^, ^173^

More research on rural populations is also needed. While only 14% to 20% of the US population currently lives in rural areas, the proportion of older‐age adults in these areas is increasing disproportionately, raising critical concerns over service and resource provisions in rural settings to support healthy cognitive aging.^174^, ^175^ Attention should be directed to conceptualization and measurement of rural contexts across a continuum of rurality relative to small town, suburban, and urban areas.^49^ A growing body of evidence indicates a high degree of interconnectedness among rural and urban areas,^174^, ^176^ and recent work suggests substantial heterogeneity among rural contexts based on the types of built and social resources.^177^ Given recent demographic shifts in rural America, the rapid growth of the older adult population, and the interdependencies of rural and urban settings, more research is needed to identify social‐environmental factors relevant to cognitive aging in rural areas.

### 5.2 Expanded conceptualizations

Cognitive aging research would also benefit from integrating more complex conceptualizations of the social environment. Otherwise, investigators may overlook novel hypotheses that run counter to widespread assumptions. For example, fast‐food restaurants are commonly^178^ but not always^179^ associated with poor nutrition and health. Yet only recently have they been identified as unique “third places” that can enhance social connectivity for certain populations.^121^, ^180^ There are also potential “dark sides” to social connectedness^181^ and benefits of solitude.^182^ For instance, while social isolation is typically linked to increased risk of morbidity and mortality, this does not appear to be true for everyone. In certain contexts, experiences of solitude, such as meditation or solo nature walks, can reduce stress and enhance cognitive function.^183^, ^184^, ^185^ These examples reiterate the importance of integrating nuance and complexity into our frameworks.

Researchers also should consider incorporating digital technologies (i.e., computers, smartphones) and online social platforms (i.e., digital environments that facilitate social interactions, e.g., social media, Zoom) into their frameworks linking the social environment to life course cognitive aging. As more and more adults age with these technologies and platforms, they will become increasingly relevant to cognitive aging across the three social environment domains. This literature is still nascent, with small localized studies, but the results suggest potential new areas of research. For example, recent work suggests a positive correlation between daily social media (e.g., Facebook, Twitter) use and emotional well‐being, particularly for older adults with smaller social networks.^186^ Others have described the use of place‐based digital platforms like NextDoor to facilitate micro‐interpersonal‐level bridging ties and meso‐level social capital within communities during times of crisis.^187^ Finally, the hybrid and remote work arrangements, facilitated by digital technology and platforms, have increased dramatically since the pandemic,^188^ with implications for work‐based social connections. For example, a recent scoping review suggests a correlation between remote work and poor psychosocial well‐being, including feelings of social disconnection and psychosocial stress.^189^

As research expands in this area, the same considerations are needed as with any other aspect of the social environment. A clear conceptual framework with measures that reflect the concept, based on the research question, will facilitate this literature as technologies and platforms likely cut across multiple ecological levels. For example, the micro‐individual‐level use of social media may be linked to the structure of one's micro‐interpersonal social network. Further, meso‐level community organizations may use social media to engage with their members and broader communities, which may facilitate meso‐level social capital and cohesion. Social media platforms themselves can be conceptualized as online third places, where people can gather to build and strengthen both bridging and bonding ties and allow for online people watching.^190^ With these new frameworks, it will be important to consider the balance of dark sides of the digital social environment, as the review on remote work arrangement suggests.

### 5.3 Expanded measures

There is also a need to develop new measures of the social environment with clearer implications for cognitive aging. For example, several critiques of popular measures of economic and racial segregation have already been published.^44^, ^45^, ^46^, ^48^ At a fundamental level, using measures that accurately reflect the theorized spatial construct is critical. When testing the role of neighborhoods, for instance, we recommend using neighborhood‐level measures (e.g., tract), rather than city‐ or county‐level measures. We also recommend using neighborhood‐level measures derived from residential data that account for spatial autocorrelation.^191^ However, even these measures do not include physical barriers that may limit access to certain types of social infrastructure, such as major roadways or waterways. New methods have been developed to account for these barriers, which may provide a clearer picture of how people navigate space in their daily lives.^192^ Others have employed resident mobility data derived from cell phones to create new measures of interconnectivity among neighborhoods and within cities. While mobility data are available only for recent years, they could help to clarify how different residential areas within cities are divided and connected.^36^, ^37^, ^193^, ^194^

Empirical research on cognitive aging and dimensions of social infrastructure, especially third and fourth places, is also nascent and based largely on detailed qualitative research. Quantitative work in this area has been mostly limited to indices that sum the number or density of specific types of places (e.g., libraries) found within certain spatial units (e.g., tracts).^195^ Measures like these are publicly available and can be linked easily to population surveys and other datasets with cognitive assessments (although a recent study calls data quality of some sources into question).^196^ However, these measures lack information on whether, when, and by whom different places are actually used. Building on insights from qualitative work, there are opportunities to develop innovative measures of social infrastructure based on high‐intensity resident mobility data, which could also be linked to cognitive health data.^197^ As one of many possible examples, mobility data could be used to create area‐level measures of foot traffic within different types of third places.

Research on social connectedness and cognitive aging has also relied on retrospective self‐reports from survey participants.^32^, ^198^, ^199^ This strategy may introduce substantial measurement error in studies of cognitive function, where a participant's memory is the very outcome in question.^98^ For these reasons, scholars are also beginning to develop new measurement strategies, such as wearable sensors that can record a participant's social interactions passively throughout the day.^200^, ^201^, ^202^ Moreover, the majority of work on social connectedness and cognitive health focuses on the effects of core networks and close ties to family and friends, while research on peripheral or bridging ties is less common. This is due, in large part, to the excessive burden associated with collecting high‐dimensional social network data and a lack of brief scales or proxy measures operationalizing access to expansive ties to neighbors, coworkers, and other community members.^24^, ^203^

## 6 CLOSING: FACILITATING RESEARCH ON THE SOCIAL ENVIRONMENT

One initiative funded by the National Institute on Aging (NIA) is focused on expanding research on a broader range of AD/ADRD risk factors. The Gateway Exposome Coordinating Center (GECC) facilitates research on the social, built, and natural environments and cognitive aging and AD/ADRD risk in multiple ways. First, the GECC convenes both leading and early‐career scholars from multiple disciplines and areas of expertise to develop conceptual, measurement, and data guidelines, including for the study of the social environment and cognitive aging. These researchers, including authors of this paper, are helping to build clarity on topics lacking conceptual unity, such as social infrastructure, third places, place‐based organizations, social capital, rurality, and digital social infrastructure.

Second, the GECC also has been developing and will soon provide access to data and measurement guidance drawn from leading researchers. For instance, our network of researchers has been developing critical reviews of the crowded measurement literature on social isolation and conceptual and data reviews of social infrastructure in relation to life course cognitive aging. Documents on recommended survey‐based measures, along with guidance on operationalization and modeling, will be provided for all major NIA‐funded datasets (e.g., HRS, National Health and Aging Trends Study). We are also creating new mobility‐based measures of socioeconomic disadvantage, affluence, and social infrastructure at multiple spatial levels. These new measures will also be linked to major NIA‐funded datasets with measures of cognitive function to help advance research on place‐based determinants of cognitive aging.

Empirical links between social environments and population health inequalities have been studied for decades, with a recent focus on cognitive aging and AD/ADRD. Although the scientific community has made major strides in understanding how the social environment might affect cognitive health, new frameworks and measures are still needed to expand knowledge in this field. The GECC is uniquely situated to advance this mission for the benefit of science and our rapidly aging population.

## CONFLICT OF INTEREST STATEMENT

The authors declare interests as outlined in the accompanying ICMJE forms. The authors declare no other conflicts of interest. Author disclosures are available in the Supporting Information.

## Supporting information




**Supporting Information**: alz71648‐Sup‐0001‐ICMJE forms.pdf
